# Addison’s disease associated with hypokalemia: a case report

**DOI:** 10.1186/s13256-021-02724-6

**Published:** 2021-03-25

**Authors:** M. Abdalla, J. A. Dave, I. L. Ross

**Affiliations:** Division of Endocrinology, Department of Medicine, Faculty of Health Sciences, Groote Schuur Hospital and University of Cape Town, Private Bag X3, J47-85 Old Main Building, Observatory, Cape Town, 7935 South Africa

**Keywords:** Addison’s disease, Hypokalemia, Tubulopathy

## Abstract

**Background:**

Primary adrenal insufficiency (Addison's disease) is a rare medical condition usually associated with hyperkalemia or normokalemia. We report a rare case of Addison's disease, coexisting with hypokalemia, requiring treatment.

**Case presentation:**

In this case, a 42-year-old man was admitted to the intensive care unit with a history of loss of consciousness and severe hypoglycemia. His blood tests showed metabolic acidosis, low concentrations of cortisol 6 nmol/L (normal 68–327 nmol/L), and high plasma adrenocorticotropic hormone 253 pmol/L (normal 1.6–13.9 pmol/L), and he was diagnosed with primary adrenal insufficiency. Surprisingly, his serum potassium was low, 2.3 mmol/L (normal 3.5–5.1 mmol/L), requiring replacement over the course of his admission. Computed tomography scan of the adrenal glands showed features suggestive of unilateral adrenal tuberculosis. Investigations confirmed renal tubulopathy. The patient responded favorably to cortisol replacement, but never required fludrocortisone.

**Conclusions:**

Coexistence of hypokalemia with Addison’s disease is unusual. We recommend investigation of the cause of hypokalemia in its own right, if it occurs with primary adrenal insufficiency.

## Background

Addison’s disease may manifest with electrolyte abnormalities, metabolic derangements, and associated endocrinopathies, some of which are reversible by replacement with glucocorticoids and mineralocorticoids alone. The clinical features of primary adrenal insufficiency are nonspecific, including fatigue, loss of weight, nausea, vomiting, and salt craving, but shock is the preeminent feature of adrenal crisis [1]. Most patients have hypotension and postural dizziness, as a result of volume depletion and loss of vascular reactivity, due to deficiency of glucocorticoids and mineralocorticoids. A cardinal sign of Addison’s disease is hyperpigmentation, which presents in nearly all patients, but the degree of hyperpigmentation is variable. It occurs as a result of a high concentration of proopiomelanocortin, a prohormone of adrenocorticotropic hormone (ACTH) and melanocyte-stimulating hormone (MSH) [2]. Vitiligo may be seen in association with autoimmune adrenal disease [3]. The most common laboratory findings include: hyponatremia, hyperkalemia, and anemia [4]. Hydrocortisone is the mainstay of treatment, and fludrocortisone is added to replace mineralocorticoid deficiency. Usually, hydrocortisone doses are divided into two or three doses, with the highest dose in the morning to mimic the circadian rhythm. Monitoring of replacement relies on clinical assessment in combination with normal sodium and potassium concentrations, along with plasma renin [5].

We encountered a classical presentation of Addison’s disease, apart from severe hypokalemia, which was an unexpected finding. We present a case of a 42-year-old man, whose workup included causes of Addison’s disease and hypokalemia.

## Case presentation

A 42-year-old man, previously healthy apart from substantial alcohol use, presented with weight loss and fatigue, and no recent history of either nausea, vomiting, or diarrhea. He was seen at his local clinic with a profoundly decreased level of consciousness. Retrospectively, we were able to elicit that he had experienced loss of libido for 4 months prior to his presentation. His Glasgow Coma Score (GCS) was 3/15, laboratory glucose was 1.1 mmol/L (normal 4.0–5.4 mmol/L), and insulin level 1.3 mIU/L (normal 2.6–24.9 mIU/L), but no blood was taken to measure C-peptide, sulfonylurea, or IGF-I levels. He received two doses of 50 mL 50% dextrose intravenously, which restored his blood glucose to 12.8 mmol/L, with a marginal improvement in his level of consciousness.

A computed tomography (CT) scan of his brain revealed no intracranial pathology. His initial biochemistry revealed a serum sodium level of 131 mmol/L (normal 136–145 mmol/L), potassium 2.3 mmol/L (normal 3.5–5.1 mmol/L), creatinine 81 µmol/L (normal 64.0–104.0 µmol/L), and ionized calcium of 1.22 mmol/L (normal 1.05–1.30 mmol/L). At the time his glucose level was 12.8 mmol/L, a random cortisol level was 6 nmol/L (normal 68 nmol/L–327 nmol/L) and simultaneous plasma adrenocorticotropic hormone (ACTH) was 253 pmol/L (normal 1.6–13.9 pmol/L), suggestive of primary hypoadrenalism, and therefore an ACTH stimulation test was not deemed necessary. His dehydroepiandrosterone sulfate of 0.9 µmol/L (normal 2.4–11.6 µmol/L) was also corroborative of primary adrenal insufficiency. In addition, thyroid functions revealed a TSH of 16.61 mIU/L (normal 0.27–4.20 mIU/L), and free T4 7.6 pmol/L (normal 12.0–22.0 pmol/L) and antithyroid peroxidase antibodies were 10 U/mL (normal <34 U/mL). His arterial blood gas demonstrated a pH of 7.22 (acidotic), PO_2_ 13.8 KPa, PCO_2_ 4.82 KPa, standard HCO_3_ 15.1 mmol/L, and base excess –12.3 mmol/L (normal –2 to +2 mmol/L), in keeping with metabolic acidosis. He was intubated for airway protection and referred to a tertiary hospital for intensive care unit management.

On examination, he was underweight but not emaciated, blood pressure was 103/62 mmHg, and he had a regular pulse rate of 98 beats per minute. He appeared clinically dehydrated and was noted to have some mild increased skin pigmentation of his palmar creases and extensor surfaces of his elbows. Cardiovascular examination was normal. Chest examination while intubated revealed no wheezes or crepitations. Examination of the abdomen was normal, and neurological review demonstrated no lateralizing signs.

In addition to mechanical ventilation, he received intravenous potassium chloride and stress doses of hydrocortisone 100 mg six hourly and thiamine. He was extubated after 24 hours of ventilation.

On normalization of his serum potassium to 3.9 mmol/L (normal 3.5–5.1 mmol/L), his serum aldosterone was less than 52.9 pmol/L (supine 49.0–643.0 pmol/L). While convalescing on a general medical ward, taking 20 mg of hydrocortisone per day and while normokalemic with potassium supplementation, investigation for his hypokalemia revealed a urinary potassium of 17.4 mmol/L (normal less than 20 mmol/L), urinary sodium of 113 mmol/L (normal less than 20 mmol/L), transtubular potassium gradient (TTKG) of 8, and urine potassium-to-creatinine [K/Cr] ratio of 3.9 mmol/mmol.

He was started on thyroxine replacement. As his blood pressure and serum potassium were normal, a decision was made to withhold fludrocortisone and stop oral potassium replacement. Investigation for renal tubular acidosis, after extubation, showed a normal arterial blood pH of 7.39 and urine pH of 6. Moreover, Fanconi syndrome was negative, including absent glycosuria, absent aminoaciduria, and urine phosphate-to-creatinine ratio of 1.22 (normal 0.20–3.14) mmol/mmol creatinine. Investigations for an underlying cause of primary adrenal insufficiency, including measurement of adrenocortical antibodies and very-long-chain fatty acids, and an intensive screen for tuberculosis, were negative. CT scan of the adrenal glands revealed a normal-size right adrenal gland, while the left adrenal gland revealed linear calcification of 827 Hounsfield units (HU), suggestive of previous tuberculosis infection. Two separate human immunodeficiency virus (HIV) tests were conducted using enzyme-linked immunosorbent assay (ELISA), both of which were negative. Considering his complaint of reduced libido, we investigated his early-morning testosterone concentrations, which were 7.7 nmol/L (normal 8.6–29.0 nmol/L), follicle-stimulating hormone (FSH) 1.8 IU/L (normal 1.5–12.4 IU/L), and luteinizing hormone (LH) 6.2 IU/L (normal 1.7–8.6 IU/L), indicating hypogonadotropic hypogonadism.

Ten days after admission, the patient was discharged from hospital well, on hydrocortisone 20 mg per day and thyroxine, as the abnormal thyroid functions persisted despite hydrocortisone. At follow-up, his oral potassium was tapered and stopped after 6 weeks, but he never required mineralocorticoid or testosterone replacement as the latter improved. On follow-up, 3 months after discharge, his urinary potassium level was 51.6 mmol/L (normal less than 20 mmol/L) and urinary Na 58 mmol/L (normal less than 20 mmol/L), suggesting that he had ongoing renal losses of potassium and sodium.

## Discussion and conclusions

We present an unusual case in which a patient with biochemical confirmation of Addison’s disease (primary adrenal insufficiency) also presented with hypokalemia, probably on the basis of a renal tubular defect. Intriguingly, this patient did not require fludrocortisone, as he was never hyperkalemic.

Investigation of this patient revealed hypokalemia, which was unexpected in the context of primary hypoadrenalism. In view of the persistent hypokalemia, we made the decision to withhold fludrocortisone. In addition, we were concerned that the patient would experience significant urinary sodium losses, which were likely to be declared on follow-up by way of low serum sodium.

Hyponatremia and hyperkalemia are major electrolyte disturbances that occur with adrenal insufficiency. Hyponatremia is mainly due to the increased release of antidiuretic hormone (ADH) [6]. Hyperkalemia in Addison’s disease is mediated mainly by hypoaldosteronism, and thus a deficiency of aldosterone will result in potassium retention, through its inability to excrete potassium in the urine [7]. However, in one series, only 34% of patients had hyperkalemia; normokalemia is, by contrast, far more common, 66% of Addison's disease patients [8], due to an aldosterone-independent regulation by the distal nephron. Secretion of potassium in the cortical collecting tubule may be mediated by the potassium-selective channels in the luminal membrane of principal cells and is the main potassium-secretory channel in the segment, which is thought to be a low-conductance inwardly rectifying channel. These channels are likely the consequence of the renal outer medullary potassium channel (ROMK) gene [9]. Similarly, in our previously published study of Addison’s disease in South Africa, we found a prevalence of normokalemia of 65%, at initial presentation [10].

We believe that the existence of hypokalemia is an unusual phenomenon in Addison’s disease, and to the best of our knowledge it has not previously been described. We suspect that the hypokalemia is likely the consequence of an additional insult that may render the kidneys unable to retain potassium. In this case, the possible cause is renal loss, due to an underlying tubulopathy, which causes either continuous or intermittent potassium losses. Renal potassium losses, particularly tubulopathies, are the likely explanation for hypokalemia, as this patient was not on thiazide diuretics, had no evidence of mineralocorticoid-induced hypertension, and, moreover, manifested with no apparent gastrointestinal losses [11]. In support of a tubulopathy was our patient’s elevated TTKG and K/Cr, as a K/Cr, value > 2.5 mmol/mmol, and TTKG value > 3 are suggestive of tubulopathy [12, 13].

Meanwhile, the metabolic acidosis that exists with Addison’s disease as a feature of aldosterone deficiency may have served to lower the potassium excretion rate in our patient’s urine [14], which could have led us to underestimate the degree of potassium loss in the urine. We also considered that his considerable alcohol consumption may have contributed to his hypokalemia, but we were unable to demonstrate either hypomagnesemia or alcoholic ketoacidosis [15]. There appears to be no evidence of genetic tubulopathy [16]. There was also no history of aminoglycoside, prostaglandin, cisplatin, and heavy-metal use to account for acquired hypokalemia [17].

There are sufficient biochemical data on which to base our diagnosis of Addison’s disease (primary adrenal insufficiency), namely hypoglycemia, profoundly low plasma cortisol, simultaneously elevated plasma ACTH, low dehydroepiandrosterone sulfate, decreased aldosterone, and metabolic acidosis [5]. We repeated the biochemical tests after commencing hydrocortisone, which reversed the metabolic acidosis and normalized the plasma ACTH concentration. Although the 21-hydroxylase autoantibodies are the most sensitive and specific for the diagnosis of autoimmune primary adrenal insufficiency, adrenocortical antibodies were measured, because of their availability, but the latter are considerably less sensitive and specific [18].

Despite the consistency the CT scan findings with tuberculosis, he had no prior history and denied ever receiving antituberculous therapy. Antituberculous therapy is known to associate with tubular dysfunction [19]. The most common extrapulmonary sites of tuberculosis include liver, spleen, kidney, bone, and adrenal glands [20]. The requirements for the diagnosis of tuberculosis (TB)-induced primary adrenal insufficiency are either a prior or current history of tuberculosis, but may also be clinically latent, by a positive Mantoux test or suggestive radiology [21]. Specific imaging characteristics that make tuberculosis of the adrenal glands more likely include size, presence of calcification, location, and contour [22]. In the early stages of adrenal tuberculosis, the adrenal glands increase in size, because of the inflammatory process, which can be detected by CT or magnetic resonance imaging (MRI). Approximately 2 years after insult, the adrenals may be normal or small because of fibrosis. The adrenal gland CT scan may show normal-sized adrenal glands or, if enlarged, mass-like with preserved contours, peripheral rim enhancement, or calcification with tuberculous adrenalitis [23]. Antiadrenal autoantibodies are usually absent in patients with adrenal TB [24].

The diagnosis of hypogonadism relies on two blood tests on separate occasions and symptoms consistent with hypogonadism [25]. It is plausible that the low testosterone levels in this patient can be explained by the gonadotropin syndrome [26]. On replacement with hydrocortisone, he experienced normalization of his libido and testosterone levels. On the other hand, we have published a series of hypogonadism occurring frequently in patients with primary adrenal insufficiency. The causes outlined in this series included excessive glucocorticoid replacement therapy; the same disease process, which led to the destruction of the adrenal cortex, may also involve the gonads or pituitary, such as tuberculosis, sarcoidosis, and histiocytosis X. Some studies have also shown an inverse relationship between cortisol concentration and release of luteinizing hormone (LH). In men, X-linked congenital adrenal hypoplasia associated with DAX-1 mutation, polyendocrine syndromes, adrenoleukodystrophy, and hemochromatosis can cause both Addison’s disease and hypogonadism [27].

Primary hypothyroidism can coexist with autoimmune primary adrenal insufficiency as part of autoimmune polyglandular syndrome (APS) [28]. Elevated TSH is sometimes seen with recovery from euthyroid sick syndrome [29]. We were unable to show an autoimmune cause for his primary hypothyroidism. It is well described that TSH may be elevated while T4 can be low in the context of cortisol deficiency, because of the crosstalk that exists between the hypothalamic-pituitary adrenal and the pituitary thyroid axes, which may be reversed with replacement [30, 31]. Although prolactin was not assessed in this patient, it is not infrequently elevated in untreated primary adrenal insufficiency, and it is reversible with replacement doses of glucocorticoids [32].

In summary, despite this classical presentation of Addison’s disease, the coexistence of hypokalemia makes it unusual. The hypokalemia could not be explained by Addison’s disease, and a careful search for an additional underlying cause was warranted.

## Data Availability

All laboratory data are available through the repository held by the National Health Laboratory Services (NHLS), South Africa, and radiology is available from the Department of Radiology, Groote Schuur Hospital, Cape Town.

## Abbreviations

ACTH: Adrenocorticotropic hormone
ADH: Antidiuretic hormone
CT: Computed tomography
TTKG: Transtubular potassium gradient
